# Health system factors influencing management of multidrug-resistant tuberculosis in four European Union countries - learning from country experiences

**DOI:** 10.1186/s12889-017-4216-9

**Published:** 2017-04-19

**Authors:** Gerard de Vries, Svetla Tsolova, Laura F. Anderson, Agnes C. Gebhard, Einar Heldal, Vahur Hollo, Laura Sánchez-Cambronero Cejudo, Daniela Schmid, Bert Schreuder, Tonka Varleva, Marieke J. van der Werf

**Affiliations:** 1 KNCV Tuberculosis Foundation, The Hague, The Netherlands; 2 European Centre for Disease Prevention and Control, Tomtebodavagen 11A, S-171 83 Stockholm, Sweden; 3 Public Health England Colindale, London, UK; 4 Ministry of Health, Social Services and Equality, Madrid, Spain; 5 Austrian Agency for Health and Food Safety, Vienna, Austria; 6 National Tuberculosis Programme, Sofia, Bulgaria

**Keywords:** European Union, Healthcare systems, Multidrug-resistance, Treatment outcome, Tuberculosis

## Abstract

**Background:**

In the European Union and European Economic Area only 38% of multidrug-resistant tuberculosis patients notified in 2011 completed treatment successfully at 24 months’ evaluation. Socio-economic factors and patient factors such as demographic characteristics, behaviour and attitudes are associated with treatment outcomes. Characteristics of healthcare systems also affect health outcomes. This study was conducted to identify and better understand the contribution of health system components to successful treatment of multidrug-resistant tuberculosis.

**Methods:**

We selected four European Union countries to provide for a broad range of geographical locations and levels of treatment success rates of the multidrug-resistant tuberculosis cohort in 2009. We conducted semi-structured interviews following a conceptual framework with representatives from policy and planning authorities, healthcare providers and civil society organisations. Responses were organised according to the six building blocks of the World Health Organization health systems framework.

**Results:**

In the four included countries, Austria, Bulgaria, Spain, and the United Kingdom, the following healthcare system factors were perceived as key to achieving good treatment results for patients with multidrug-resistant tuberculosis: timely diagnosis of drug-resistant tuberculosis; financial systems that ensure access to a full course of treatment and support for multidrug-resistant tuberculosis patients; patient-centred approaches with strong intersectoral collaboration that address patients’ emotional and social needs; motivated and dedicated healthcare workers with sufficient mandate and means to support patients; and cross-border management of multidrug-resistant tuberculosis to secure continuum of care between countries.

**Conclusion:**

We suggest that the following actions may improve the success of treatment for multidrug-resistant tuberculosis patients: deployment of rapid molecular diagnostic tests; development of context-specific treatment guidance and criteria for hospital admission and discharge in the European context; strengthening patient-centred approaches; development of collaborative mechanisms to ensure cross-border care, and development of long-term sustainable financing strategies.

**Electronic supplementary material:**

The online version of this article (doi:10.1186/s12889-017-4216-9) contains supplementary material, which is available to authorized users.

## Background

Tuberculosis (TB) remains a major global health problem with an estimated 9.0 million people that developed TB and 1.5 million that died from the disease in 2013 [1]. Since the World Health Organization (WHO) declared tuberculosis a global public health emergency in 1993 progress has been made with falling TB incidence and mortality rates over the last decade [1]. An area of great concern, however, is the spread of *Mycobacterium tuberculosis* strains resistant to anti-TB drugs [2]. One serious type of drug resistance, multidrug-resistant tuberculosis (MDR-TB), is defined as TB caused by strains resistant to the two most powerful first-line TB drugs, isoniazid and rifampicin. It is estimated that one fifth of the MDR-TB global burden is in the European Region of the WHO [1]. European Union (EU) and European Economic Area (EEA) countries reported 1484 cases of MDR-TB in 2013 [3]. The reported notification rate of MDR-TB in EU/EEA countries has slightly declined since 2007.

**Fig. 1:**
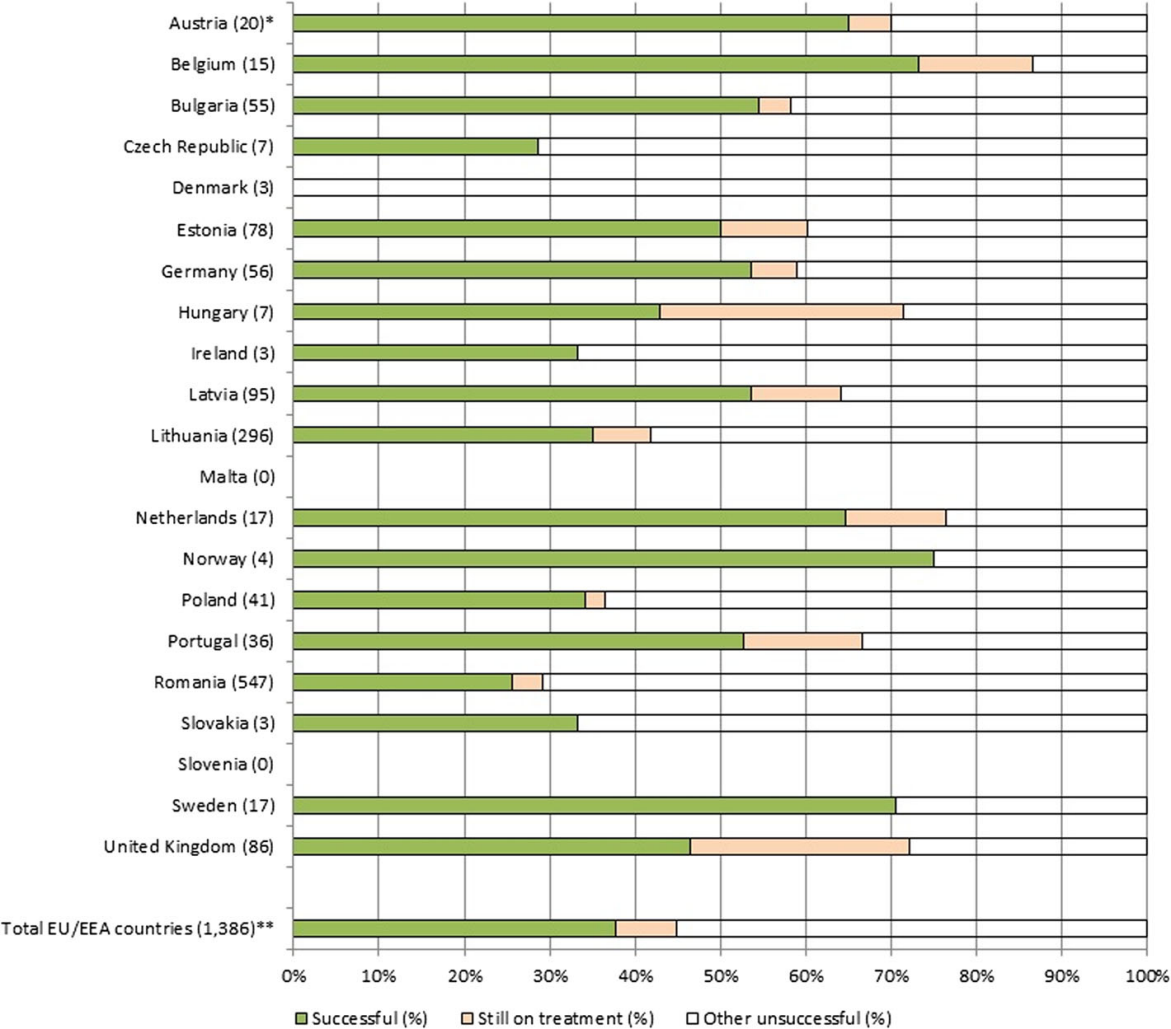
Multidrug-resistant tuberculosis treatment outcome in European Union and European Economic Area countries after 24 months of 2011 cohort [3]. EEA = European Economic Area; EU = European Union. Successful refers to the combined treatment outcome categories cured and completed; other unsuccessful refers to the categories death, failure, lost to follow-up, still on treatment and not evaluated (transferred out and unknown). * In brackets the number of notified multidrug-resistant tuberculosis cases in 2011. ** Cyprus, Finland, France, Greece, Iceland, Italy, Liechtenstein, Luxembourg and Spain did not report treatment outcome of the 2010 cohort of multidrug-resistant cases

According to the latest tuberculosis surveillance and monitoring report in Europe, the treatment success rate in EU/EEA countries was 38% for all MDR-TB cases notified in 2010 and 47% for new culture-confirmed pulmonary MDR-TB cases, at 24 months evaluation [3]. This is far below the target of 70% among new pulmonary MDR-TB cases set in the Framework action plan to fight tuberculosis in the European Union [4, 5]. Globally, 48% of MDR-TB cases who started treatment in 2011 successfully completed [1]. Although, the assessment of treatment outcome in the EU/EEA also includes patients that are not put on second line treatment, treatment success rates are poor compared to the global outcomes. Of the EU/EEA 2011 cohort, 17% of all MDR-TB patients died, 16% had been classified as treatment failure, 19% of patients were lost to follow-up, 7% of patients were reported as still on treatment and 3% had transferred out or had an unknown result after 24 months [3]. Only three out of 21 EU/EEA countries that report on MDR-TB treatment outcome achieved a success rate of ≥70% of all MDR-TB cases; seven had a successful treatment outcome rate of 50‑69% and in nine countries less than 50% of MDR-TB patients successfully completed treatment; two countries reported MDR-TB treatment outcome but had 0 cases in 2011 (Fig. 1). Also, nine EU/EEA countries did not report MDR-TB treatment outcome data for the 2011 cohort.

The treatment of MDR-TB is complicated by a long duration, with a minimum length of 20 months, and more toxic and less-effective medication than drug-susceptible TB [6]. Socio-economic factors and patient factors such as demographic characteristics, behaviour and attitudes can lead to unfavourable treatment outcomes [7–9]. Cases with an unfavourable treatment outcome may give rise to further transmission of MDR-TB and they are at risk of developing extensive drug resistance (XDR) [10].

The importance of health systems in achieving desired public health outcomes is well recognized. Though health systems are highly context-specific, they share certain characteristics that are vital for achieving good system performance and satisfactory results in the delivery of population health, such as sufficiently trained and motivated healthcare workers and sustainable financing systems [11]. Failure or success of health systems to deliver desirable health outcomes can be influenced by different factors [12]. This study was conducted in four EU/EEA Member States to identify and better understand the role that various characteristics of a healthcare system play in achieving a good outcome of MDR-TB treatment.

## Methods

### Country selection and selection of persons for interviews

We selected four EU countries for the case studies based on the following criteria: a) at least 20 notified MDR-TB cases in 2009 [13]; b) geographical location of the countries in the EU (one central, one eastern, one southern and one western EU country); and c) different treatment success rates of the 2009 MDR-TB cohort (i.e. high (> 60% treatment success), low (< 50% treatment success) or unknown). The selected countries were invited to participate in the study and all agreed.

The objective was to interview representatives from all key stakeholder groups of the health system having direct or indirect impact on MDR-TB treatment. We grouped the stakeholders in three categories: policy and planning authorities (ministry of health, national institutes for public health, national TB programmes); healthcare providers in outpatient, inpatient, and long term care facilities; and civil society organisations involved in service delivery and patient support (charity or other relevant non-governmental organisations). The individuals invited for the interviews were selected by the national TB programmes contact persons, designated by each participating country for the purposes of this study (quota sampling). All four countries provided a letter that the study was not subject to an ethical review, because it involved interviewing stakeholders and not patients. The letters were provided by the ethical committees of the following organisations: Österreichische Agentur für Gesundheit und Ernährungssicherheit GmbH (Austria); Specialized hospital for active treatment of pulmonary diseases in Gabrovo (Bulgaria); Instituto the Salud Carlos III, Fundación Centro National de Investigaciones Oncológicas Carlos III, Fundación Centro Nacional de Investigaciones Cardiovasculares Carlos III (Spain); and Public Health England (United Kingdom). Nevertheless, all interviewed individuals provided their oral agreement to participate in the interviews and either oral (via phone) or written (via e-mail or sms) agreement to be listed in the acknowledgement section.

### Questionnaire and interviews

**Fig. 2:**
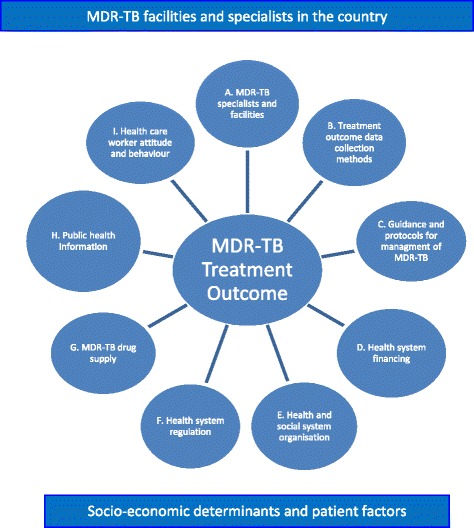
Conceptual framework of components of health system influencing MDR-TB treatment outcome

A guide for semi-structured interviews was developed for the interviews with the different categories of stakeholders of the healthcare system (Additional file 1). Questions in the guide were formulated according to a conceptual framework of components of health system factors influencing MDR-TB treatment outcome identified by the researchers (Fig. 2). The guide was tested in Belgium, an EU country not included in the study. The goal of the pilot test was to assess construct validity. The pilot made clear that the pre-test questions related to recording of clinical and social risk factors would not address the objective of our study. Firstly, because these risk factors, such as HIV-co-infection, diabetes mellitus, homelessness and drug use, are also risk factors for normal sensitive TB and often already recorded in the surveillance systems. Secondly, because our study did not intend to collect data and measure the extent of these factors for MDR-TB and MDR-TB treatment outcome. These questions were deleted and instead, we requested the national TB programme contact persons to provide us with published and/or unpublished studies on MDR-TB and MDR-TB treatment outcome in their countries before the visits. After piloting and revision, the questionnaire was translated into Spanish and Bulgarian. The questionnaire contained nine domains related to the components in the conceptual framework: a) MDR-TB facilities and specialists in the country; b) treatment outcome data collection methods; c) available guidance and protocols for management MDR-TB patients; d) health system organisation and financing; e) health and social system organisation; f) health system regulation with regard to TB and MDR-TB treatment; g) availability and supply of MDR-TB drugs; h) public health information approaches for prevention and control of MDR-TB; and i) behaviour and attitude of healthcare workers towards MDR-TB treatment. Representatives of the different categories of stakeholders were asked the same set of questions in all four countries; 41 out of the total of 57 questions were intended for more than one stakeholder category. Additionally, there were three open questions posed to the interviewees on their opinion regarding main health system factor(s) contributing to success, shortfalls and possible areas for improvement in the MDR-TB treatment. Interviews were conducted face-to-face (except two conducted by telephone, because of large travelling distance) by two researchers (all medical doctors). All interviewees were asked to fill in most of the questionnaire before the visit to facilitate in-depth discussion during the interview using probing questions. Questions regarding sensitive legal and financial issues, behavioural practices and attitude were only addressed at the end of the interview. The target was to obtain at least one representative from the three groups of stakeholders. The interviews were carried out in the first quarter of 2014. In Bulgaria and Spain, the national TB programme contact person helped with interpretation during the interview when needed.

### Data analysis

One of the researchers compiled a summary report of the questionnaire per country which was reviewed by the other researcher. The responses obtained for nine domains were analysed following the six building blocks for health systems from ‘Strengthening health systems to improve health outcomes: WHO’s framework for action’: service delivery; health workforce; information; medical products, vaccines and technologies; sustainable financing and social protection; leadership and governance [11]. Table 1 shows which question provided input for a building block. Information from the open questions and obtained during the interviews was used to identify good practices from each country. The draft results were shared with the national TB programme contact person to validate the findings.

**Table 1:** Relation between the questions organised in nine domains of the guide for questions to the six building blocks for health systems

	A. MDR-TB facilities and specialists in the country	B. Treatment outcome data collection methods	C. Available guidance and protocols for management MDR-TB patients	D. Health system organisation and financing	E. Health and social system organisation	F. Health system regulation with regard to TB and MDR-TB treatment	G. Availability and uninterrupted supply of MDR-TB drugs	H. Public health information approaches for preventions and control of MDR-TB	I. Behaviour and attitude of healthcare workers towards MDR-TB treatment
Country’s health system organisation and MDR-TB situation	1,8			32					
Service delivery									
-Diagnosis of MDR and XDR-TB			21–23						
-MDR-TB treatment			25,26						
-(Duration of) Hospitalisation	2–4,9								
-Teamwork and multidisciplinary teams			24,28–30						
-Cross-border MDR-TB case management									
Health workforce	5,6,10		31		40–46				56–57
Health information		13–20							
Medical products, vaccines and technologies			27				50–54		
Sustainable financing and social protection				33–39					
Leadership and governance	7,11					47–49		55	

The letters in the columns refer to the number of the question in the Guide for questions (see Additional file 1)

## Results

In the four countries, altogether 35 persons were interviewed (Table 2).

**Table 2:** Number of interviewees by country and by stakeholder group

	Representatives of policy and planning authorities	Healthcare providers	Persons representing civil society organisations	Total
Austria	2	7	0	9
Bulgaria	3	2^*^	3	8
Spain	4	5	0	9
United Kingdom	3	5	1	9
Total	12	19	4	35

^*^The interview with the prison medical staff was counted as one, since this was a group interview

The healthcare systems in Austria (AT), Bulgaria (BG), Spain (ES) and the United Kingdom (UK) varied from a centralised governance structure to an autonomous regionalised one; with different financing structures from general taxation to national health insurance schemes. One country (Bulgaria) received support from the Global Fund to Fight AIDS, TB and Malaria (the Global Fund) for TB prevention and control.

Below we report the results by building block for health systems.

### Service delivery

#### Diagnosis and treatment

Rapid molecular diagnostic tests for diagnosis of MDR-TB were not available in some hospitals and criteria for use were lacking or not known in three of the four countries (AT, ES, and UK; BG had criteria for use of line probe assays). One pulmonologist mentioned: *“Rapid molecular tests are not available in my hospital and drug-susceptibility test results can take up to 2 weeks for TB and 4 weeks for MDR-TB, which is not timely”.* Occasionally, the time between the initial diagnosis of TB and the MDR-TB diagnosis could be as long as 4–5 months in one country (BG), this was in a patient not eligible for molecular testing according to protocol and with a long delay in obtaining results from conventional drug-susceptibility testing.

The responses also indicated that very often clinicians were unable to treat MDR-TB patients with injectable second-line TB drugs for the recommended duration because of development of severe adverse events. Clinicians were also inclined to treat and adjust MDR-TB treatment based on professional insights and experience and recent peer-review publications. Also, newer second-line drugs, such as linezolid, were more widely used in MDR-TB treatment regimens in three countries (AT, ES, and UK) than currently recommended by the WHO guidelines [6, 14].

#### Hospitalisation

MDR-TB patients were frequently hospitalised for controlled initiation of treatment and preparation for treatment adherence after discharge. Practices on when to discharge MDR-TB patients varied widely and in none of the countries guidance was available on the optimal duration of hospitalisation and criteria for end of hospitalisation.

Two countries (AT and BG) had a TB hospital ward where about 80–90% of the country’s MDR-TB patients, 15–40 new MDR-TB admissions annually, start MDR-TB treatment. In these two countries, either the hospital continued monitoring patients intensively after discharge or an MDR-TB Expert Committee monitored the ambulatory treatment. By contrast, in the other two countries (ES and UK), in-patient MDR-TB treatment was performed by many hospitals with clinicians hospitalising and treating about 1–5 cases annually and providing follow-up care for these patients during ambulatory treatment.

In Austria and the United Kingdom some clinics have introduced a comprehensive hospital discharge plan for each MDR-TB patient including information on management of clinical condition(s), information on housing facilities, a plan for management of the patient in the outpatient situation and infection control measures.

#### Multidisciplinary teams and MDR-TB expert committees

In Bulgaria, an MDR-TB Expert Committee decides on the management of all MDR-TB patients, while in Spain a regional MDR-TB Committee has been established. Such committees may initiate treatment (choice of regimen), support the management of side effects, monitor treatment progress, compliance, and regimen changes, and decide or advice on the end of treatment. In the United Kingdom, an internet-based MDR-TB Clinical Advice Service is established [15] that gives clinical advice on MDR-TB case management to professionals who voluntarily contact the service. In all countries, in-hospital teams formally discussed management of MDR-TB in-patients, often on a weekly basis. The importance of this collaboration was emphasized by one of the pulmonologists, stating: “*Short courses are organized for physicians and nurses, but probably the most important source for training is the medical literature and weekly clinical meetings.”*

Countries had different approaches to monitor patients during ambulatory care. In some instances, this was performed during monthly evaluation meetings of the MDR-TB Expert Committee, in others via multidisciplinary team meetings or consultation room discussions between the medical doctor, the patient and the nurse accompanying the patient.

#### Cross-border MDR-TB case management

One country (AT) faced an increase in MDR-TB patients from other EU and non-EU countries. These patients seek treatment outside their country, because of better treatment options elsewhere. A nurse expressed: *“The word goes around in the migrant communities that MDR-TB can be treated in this country/hospital, and it attracts new patients”* and one physician explained: *“Some migrants come with all the papers and the diagnosis MDR/XDR-TB to get treatment here, because second-line drugs are not available in their countries”.* Foreign-born patients diagnosed with MDR-TB in an EU country, e.g. students or workers from other EU or non-EU countries, frequently return to their home country during the lengthy MDR-TB treatment. Three countries (AT, ES and UK) participating in the study reported serious difficulties in ensuring continuation of treatment for migrating patients. Moreover, according to the EU case definition the migrated cases are reported as ‘transferred out’ and thus the surveillance system classified them as unsuccessful treatment outcome. Nevertheless, according to the interviews, medical professionals put much effort into close monitoring of patients that migrate during MDR-TB treatment and provided support for the continuation of their treatment, whenever possible, either by email or other means of communication. One nurse explained: “*My MDR-TB patient had to return to India and contacted me after arrival. We continued having email contact until the end of her treatment. She even sent me the results of the monitoring of her treatment. After completion of treatment she could be classified as “completed treatment” but the system only allows “transferred out”.*

### Health workforce providing patient-centred services

In all settings in the four countries, nurses (community or hospital-based) or social workers were assigned as case managers during the ambulatory care of MDR-TB. They provided psychological and social support tailored to patient needs. Healthcare professionals interviewed in all four countries were very committed. One of the interviewees mentioned pride, engagement with and dedication to their work, as a relevant attitude of the professional worker to support these often difficult to treat patients. Patients’ appreciation of support given by nurses was phrased as: “*In the beginning patients feel angry or annoyed, because their privacy is gone, but at the end they miss the contacts and may cry*”.

### Health information

MDR-TB treatment outcome data were collected through TB surveillance systems. One of the participating countries (ES) recently changed the surveillance system to enable recording and reporting the treatment outcome of MDR-TB patients. One country (UK) performed quarterly reviews of all MDR-TB patients on treatment. The low treatment success rate reported in one country (BG) was due to long delays in starting MDR-TB treatment, due to initial absence of MDR-TB drugs, leading to high death rates and loss of follow-up before treatment was started, and slow culture methods. In two countries (AT and UK), some patients were still on treatment after 24 months, or transferred or with unknown treatment outcome (e.g. due to migration). An interviewee of a surveillance unit responded*: “If outcome data are missing in the national reporting system, our department contacts the relevant local public health authority to collect the missing data”.* In Spain the area epidemiologist meets with medical doctors in the hospital to discuss TB patients every 3‑6 months.

### Medical products, vaccines and technologies

None of the respondents in the four countries reported challenges with availability of laboratory consumables, drugs for treatment of MDR-TB, or the procurement process for MDR-TB drugs. Bulgaria had started MDR-TB treatment in 2009 with Global Fund support. In Bulgaria the management of MDR-TB drugs was centralised (incl. Distribution of drugs to the penitentiary sector and for ambulatory care). This country used MDR-TB patient drugs kits that followed the patient irrespective of the place of treatment. In the other three countries it was the responsibility of (hospital) pharmacies to procure MDR-TB drugs.

### Sustainable financing and social protection

The budget for (MDR-) TB treatment and care was centralised and ear-marked in three of the four countries (AT, BG and ES) in which we performed the study. The practical arrangements differed, but in essence, state health insurance or regional or central government budgets covered the costs of MDR-TB care. In the UK the allocation of funds was determined by decentralised groups, though this arrangement was debated with discussion as to whether funding for MDR-TB treatment and care should be centralised and ear-marked in the future. Support from the Global Fund was essential for MDR-TB treatment in Bulgaria. Respondents raised concerns about the sustainability of MDR-TB treatment when this external funding would stop.

Healthcare providers in two countries (AT and UK) reported inadequate funding for in-patient care. One pulmonologist stated: “*Hospitals receive a maximum fixed tariff to hospitalise TB patients, but there is no special tariff for MDR-TB patients. Hospitalisation of these patients costs about ten times more”*. Even though concerns were expressed, services were continuously available and have not been interrupted so far.

All four countries provided free treatment for MDR-TB patients, including for uninsured or undocumented migrants. Different forms of legislation guaranteed this free MDR-TB treatment, which in some instances also covered transportation to receive daily intravenous MDR-TB treatment, or even daily food vouchers. Social support was in place but often limited to the country’s own citizens or EU citizens. One nurse stated: “*There is not a clear policy on enablers for MDR-TB patients. It can differ for each health facility that submits a request for an enabler packages.”*

### Leadership and governance

In all four countries, close collaboration with drug substitution services and HIV services was established. In the prison sector, TB screening and referral arrangements for MDR-TB patients were in place in three countries (AT, ES and UK), while Bulgaria had a dedicated TB (incl. MDR-TB) clinic in the prison, with well-established communication lines with the MDR-TB hospital and pharmacy, and the ministry of health authorities. In Bulgaria, several nongovernmental organisations developed community-based initiatives to raise community awareness, case-finding and treatment support, particularly among hard-to-reach individuals in these communities.

Nongovernmental organisations, in Spain contracted by the government, were also involved in providing support for care (e.g. providing DOT services, housing for MDR-TB patients, support in obtaining work, and support for migrants). Collaboration and involvement of non-government sector was seen as an indirect contribution for obtaining good treatment outcomes. The respondent of a nongovernmental organisation mentioned: “*They [community workers] support patients, provide information, motivate adherence, give food support, observe drug intake (only for latent TB infection and TB, not for MDR-TB), and assist with the national pension request*”.

Selected experiences and good practices from each country are provided in Table 3.

**Table 3:** Selective experiences and good practices in four selected European Union countries in treating MDR-TB patients successfully

Austria
1. Initial hospitalisation of MDR/XDR-TB patients during the intensive phase of treatment in an environment conducive for long term admission, including intensive physiotherapy, sports and leisure activities. In-patient management of MDR-TB patients is concentrated in a limited number of health facilities.
2. Patient-centred care with drug-substitution for illicit drug users, antiretroviral treatment for HIV-infected MDR/XDR-TB patients and attention for other medical conditions.
3. In Vienna, social workers of the municipality provide intensive support to hospitalised (MDR/XDR)-TB patients, prepare for discharge and the ambulatory care and continue support during the ambulatory phase, including directly observed treatment.
4. Free TB treatment, exempting TB patients to pay the compulsory food contribution fee during admission and the general fee for drug prescription, and the ministry of health covering the cost of TB treatment for undocumented migrants.
5. Use of data at level of collection and monitoring of treatment outcome by the MDR-TB specialist.
Bulgaria
1. The Global Fund supported the TB programme with establishing a case-based TB register (in place since 2007), accreditation of the National Reference Laboratory, development of guidelines (including MDR-TB guidelines), renovation of TB wards, improvement of infection control and procurement of MDR-TB drugs (available since September 2009).
2. National MDR-TB Expert Committee deciding on treatment of all MDR-TB patients, follow-up of all patients during the whole treatment, including patients in the prison sector.
3. Prison health staff and social workers educating TB patients in the prison as well as ensuring continuation of care for prisoners with TB moving to another prison in the country. After release, patients are directed to the regional health facility of their region of residence with a stock of drugs for 5 days. A document accompanies the patient that needs to be signed by the regional TB coordinator to state that the patient has reported in the region, and is returned to the prison health authorities to ensure that TB treatment is continued.
4. Nongovernmental organisations, with representatives from the hard-to-reach populations, raising community awareness, doing active case finding and supporting TB and MDR-TB case management. In-patient nurses working outside of the hospital providing ambulatory support, including directly observed treatment to MDR-TB patients.
5. Centralized management of MDR-TB drugs, including storage and disbursement, organized through one pharmacy.
Spain
1. Strong interest of autonomous regions and national minister of health in public health that resulted into legislation for free preventive services and also free treatment for TB and other diseases, also for uninsured undocumented migrants. (MDR)-TB patients exempted from the out-of-pocket contributions for drugs.
2. Multidisciplinary consilium in place in some autonomous regions that decides on MDR-TB treatment of patients, e.g. the “Galician Consilium for the assessment of treatment of resistant TB cases”.
3. Red Cross nurses providing directly observed treatment as a contracted service in the autonomous region of Madrid accompany patients into the hospital consultation room during ambulatory follow-up visits.
4. Vigorous monitoring of side effects in patients on MDR-TB treatment in the hospitals and adjustment of treatment as needed (e.g. due to ototoxicity of aminoglycosides).
5. Well-organized referral system from prisons to the primary care physician with involvement of the Surveillance Unit in the autonomous regions.
United Kingdom
1. Hospital nurses continue to visit the patients at home after discharge from hospital.
2. Well-planned discharge from hospitals of MDR-TB patients (hospital discharge plan).
3. MDR-TB patients receive different kinds of social support, including housing, and bed and breakfast arrangements.
4. MDR-TB Advisory Group provides advice to clinicians treating MDR/XDR-TB patients in the country.
5. A project with a faith-based organisation providing housing to Eastern European TB and MDR-TB patients and assisting them to find work.

## Discussion

This study assessed the contribution of health system components, in four purposively selected EU countries, to the treatment results of MDR-TB patients. MDR-TB patients face several challenges to complete their treatment. By definition, the bacteria are resistant to the most effective drugs. Less effective, more toxic second-line drugs have to be taken during almost 2 years by patients that often live in vulnerable situations. Thus next to health system factors, patient factors are important to successfully overcome the disease. Patient factors that have been reported to be associated with an unsuccessful multidrug-resistant treatment outcome are for example older age, alcohol abuse, and HIV infection [16]. Our analysis focussed on health system factors.

Health services in the four EU countries were generally well equipped to diagnose and treat MDR-TB, although availability and use of rapid molecular tests differed between hospitals and countries. The European Union Standards of TB Care recommend that these tests should be performed in settings or populations in which MDR-TB is suspected in a patient [17]. The long delays in laboratory diagnosis in MDR-TB patients observed in one country (BG) in our study indicate that countries could benefit from monitoring and analysing an indicator of time to MDR-TB laboratory diagnosis in their surveillance system to support more rapid laboratory diagnosis of drug resistance.

Interviewed clinicians often treated MDR-TB patients with an individualised treatment regimen different from internationally advised schemes [6], as was also reported before in a study in EU/EEA countries [18]. The evidence-base of choice of drugs and optimal length of MDR-TB treatment in international guidelines is of very low quality and availability of new MDR-TB drugs is changing the therapeutic field rapidly [19]. Furthermore, most EU/EEA countries have facilities, funds and dedicated staff to treat and monitor the relatively few MDR-TB patients intensively, including use of novel technologies such as therapeutic drug monitoring [20, 21]. Thus, it is understandable that in these settings the latest evidence, that has not yet reached the international guidelines, and newer, often more expensive drugs are applied.

Initial hospitalisation of MDR-TB patients was considered important by most respondents in our study to successfully treat MDR-TB patients. These views seem in contrast with the plead for ambulatory TB care services in many Eastern European and MDR-TB high burden settings [22], which is supported by evidence that MDR-TB treatment outcomes of hospital treatment or ambulatory treatment models are similar with reduced costs for patients and the health system in the ambulatory care model [23, 24]. Although ambulatory care has advantages, hospitals are needed to isolate infectious MDR-TB patients and to initiate treatment, since this requires specific knowledge and skills of the healthcare workers [14]. Also, the number of MDR-TB patients in the EU countries included in our study is relatively small which makes short-term hospitalisation feasible.

All settings in the four countries had a case manager assigned to MDR-TB patients to support patients during the lengthy treatment. Nurses play a critical role in early detection of side effects and adherence of MDR-TB treatment [25]. A review on the effectiveness of nurse case managers in three major chronic diseases suggested that this approach had the potential to achieve better health outcomes for patients with long-term conditions [26].

Three of the four studied countries faced challenges in completing treatment in foreign-born MDR-TB patients because they migrated, or were forced to leave the country, during treatment. A Wolfheze consensus statement advised a minimum package for cross-border TB control and care, and stated that these patients should be allowed to complete treatment in the European host country irrespective of legal status, and if they travel to another country then continuity of care should be ensured [27]. In the revised WHO definition of MDR-TB treatment outcome ‘transferred out’ to another unit is now classified as ‘not evaluated’ [14]. However, since migrating MDR-TB patients during treatment may frequently interrupt or discontinue therapy, efforts should be made to document the treatment outcome of these patients. This is an area for further research and for assessing good or bad practices.

Centralised funding schemes with ear-marked and protected budgets for (MDR-) TB treatment and care, including those without insurance or stay permits, was seen as a key favourable factor for the successful treatment of MDR-TB. Even though the organisation of the financial systems differed in the four countries, overall availability of sufficient financial resources was reported. Concerns and uncertainties existed about the sustainability of MDR-TB treatment in Bulgaria receiving a significant contribution of the Global Fund to the budget for MDR-TB control. Some concerns were also expressed when it comes to social support, which was not accessible to all groups of migrants in all settings. Social support for MDR-TB patients is fundamental for a patient-centred approach and to ensure treatment adherence [14].

Involvement of nongovernmental organisations and civil society organisations in (MDR-)TB prevention, diagnosis, treatment and care is considered essential because they know the local context and have the ability to engage marginalized groups [28]. Their collaboration could greatly enhance treatment outcomes [29], as was also stated by respondents in our study.

A potential bias of our study is that countries, institutions and persons participating in the interviews had an explicit interest in the topic. The study results thus may have an over-representation of enabling factors and best practices. Furthermore, we included participants from capital cities for all countries, but we were not able to access regional institutions in all countries. Therefore, especially in Spain and United Kingdom, where regions have significant autonomy in providing health care, we may have missed important supporting or obstructing factors since regional experiences and opinions may differ from those found in the capital city or in one specific highly-populated region. Our study focused on the effect of health system factors on MDR-TB treatment outcomes. We did not collect information on other important factors with a potential impact on MDR-TB treatment outcome such as socio-economic and patient-related factors.

We planned to include civil society organisations in our study. However, only two countries (BG and UK) had a civil society organisation working on TB so our findings do not represent the perspective of civil society organisations in all four countries. Another important limitation is that we did not interview MDR-TB patients, because the study design and protocol did not allow for interviewing MDR-TB patients. Thus, we miss their perspective on and experience with the MDR-TB treatment process.

Health systems are complex and we tried to structure our analysis by using a conceptual framework. We used the model of the six building blocks to distinguish health system components. The ‘service delivery’-building block was over-presented in our study, probably because of the complexity of MDR-TB. Other functions of the health systems, however, are equally important in achieving good MDR-TB treatment results. The challenge is to identify those components of the health system that need strengthening. In 2014, WHO developed the End TB Strategy which has three pillars and ten components [30]. Pillar 1 (Integrated, patient-centred care and prevention) addresses issues of the ‘service delivery’-building block, the other five building blocks fit more within pillar 2 (bold policies and supportive systems). While it is useful to distinguish parts of a health system, it is important to recognize the inter-dependency between building blocks, pillars or components, and the need for a more integrated response towards disease management.

## Conclusion

The research carried out in the context of this initiative in the four EU countries identified the following healthcare system factors that are key to achieving good treatment results for patients with MDR-TB: timely diagnosis of drug-resistance; supportive financial systems; patient-centred approaches; intersectoral collaboration; motivated and dedicated healthcare workers; and well-established cross-border management of MDR-TB.

Based on our findings we suggest that EU/EEA countries consider the following actions to arrive at better MDR-TB treatment success rates: i) deployment of rapid molecular diagnostic tests to ensure timely diagnosis of MDR-TB when MDR-TB is suspected; ii) development of guidance on treatment regimen design for the European context that includes the latest scientific evidence and that is tailored to the EU setting; iii) development of European criteria for hospital admission and discharge, and ambulatory management of MDR-TB patients; iv) further strengthening of patient-centred approaches encompassing the social needs of MDR-TB patients and sharing successful examples between countries; v) development of collaborative mechanisms to ensure a continuum of care between countries, including cross - border reporting of treatment results; and vi) ensure sustainable financing for prevention, diagnosis, treatment and care for MDR-TB patients.

## Additional file

Additional file 1: ANNEX Guide for questions. (DOCX 43 kb)

## Abbreviations

DOT: Directly observed therapy
EEA: European Economic Area
EU: European Union
MDR-TB: MultidrugMultidrug-resistant tuberculosis
TB: TuberculosisTuberculosis
WHO: World Health Organization
XDR-TB: Extensive Extensive drug resistance tuberculosis
