# Supplementary material for: Health system factors influencing management of multidrug-resistant tuberculosis in four European Union countries - learning from country experiences
Source: BMC Public Health. 2017 Apr 19;17:334. doi: 10.1186/s12889-017-4216-9 (PMC5395777; doi:10.1186/s12889-017-4216-9)
Supplement: Additional file 1: — ANNEX Guide for questions. (DOCX 43 kb) [file 12889_2017_4216_MOESM1_ESM.docx]

# ANNEX Guide for questions

## Part 1

1. MDR TB facilities and specialists in the country
2. Please check MDR TB treatment outcome in the country (Per country a table with pre-filled data of MDR TB cases and treatment outcome according to ECDC/TESSy was provided).
3. Please provide information about the number and distribution of hospitals/health facilities treating MDR TB patients in regions of the country.
4. Which additional services are in place for hospitalized MDR TB patients and to which extent are they used by the MDR TB patients? (e.g. physiotherapy, nutritional support, psychological care, leisure and sport activities)
5. How many new cases are treated annually in MDR TB treating health facility/facilities (based on an average over 5 years)?
6. How many specialists treat MDR TB patients?
7. What type of specialist are these?
8. Describe the mechanisms of collaboration (if any) between the various programs/specialists. (e.g. collaboration with HIV specialists, methadone programmes) (probing)
9. What is the average length of treating MDR-TB patients? (based on an average of 5 years)
10. What is the average length of hospitalization of a MDR-TB patient? (based on an average of 5 years)
11. To whom do MDR TB treating facilities refer cases for ambulatory treatment and how is this organized? (probing)
12. How is treatment of MDR TB patients continued in the prison system if a MDR TB patient on treatment is detained and how is treatment continued after release? (probing)
13. How is treatment of MDR TB patients in asylum seekers organized and how is treatment continued after an asylum seeker leaves a centre for permanent residence in the country? (probing)
14. Treatment outcome data collection methods
15. Who enters the MDR TB treatment outcome data in the National TB Register (at local level)?
16. Are there any specific checks (at national level) to assess completeness of MDR TB outcome data?
17. Are there any data entry checks done to ascertain accuracy of treatment outcome data?
18. Can you describe the mechanisms that exist to collect missing MDR TB treatment outcome data or correcting these data? (probing)
19. When are assessments of MDR TB treatment outcome data done?
20. Have you performed an analysis / do you collect information at national level to identify what risk factors are associated with “inadequate” treatment outcome in MDR TB patients? (probing)
21. What was the result?
22. What has been done with the results? (probing)
23. Available guidance and protocols for management MDR TB patients
24. Are rapid molecular diagnostic tests (e.g. Line Probe Assays (Genotype ®) or Xpert MTB/RIF (GeneXpert ®) applied?
25. Is MDR TB treatment generally started directly (within 2 days of test result becoming available) after molecular diagnostic tests show rifampicin and/or isoniazid/rifampicin resistance?
26. When do you receive the DST results from the laboratory for MDR TB patients after positive culture? Do you consider this timing adequate or does it delay the treatment? (probing)
27. Who decides on the MDR TB treatment regimen of an individual patient?
28. Which protocols/algorithms are currently used for MDR TB treatment?
29. Can MDR TB treatment start directly after (phenotypic) confirmation? Do criteria exist for priority setting among treating MDR TB cases?
30. Are all Second Line Anti-TB medicines, listed in the national TB guidelines, always available?
31. Who decides on the end of MDR TB treatment?
32. How is this communicated to the TB public health services?
33. Is there sharing of expertise of treating MDR TB patients, e.g. in a consilium? (probing)
34. What MDR TB education and training the MDR TB treating specialists/nurse received over the last 5 years?
35. Health system financing
36. Describe shortly the organization of the MDR TB services and how the different departments/institutions communicate at the national level (MOH, PHI) and at the local level (HCF, Patient Organization) (probing)

In-patient care

1. By whom and how is in-patient treatment and care for MDR TB patients financed?
2. Explain how medical specialists treating MDR TB patients are remunerated (probing)
3. Are there specific incentives / payments for specialists to ensure successful MDR treatment outcome?
4. Are there patient fees or other financial charges for in-patient MDR TB treatment and care?
5. Are there specifics in the financing system that may jeopardize adequate treatment of MDR? Please explain

Out-patient / ambulatory care

1. Explain how the costs of ambulatory care are financed? (probing)
2. What kind of financial incentives or other enablers (if any) are provided to the MDR TB patients during ambulatory care to comply with treatment?
3. Health and social system organization
4. Describe the ambulatory treatment support system to MDR TB patients (probing)
5. What instruments are used to monitor patient compliance?
6. When and what action is undertaken when the patient does not come to an appointment or the person providing DOTS finds out that the patient does not take its medicines? (probing)
7. Is staff/transport available to visit patients at home?
8. How frequently are MDR TB patients monitored by the treating physician?
9. How is the referral of patients on MDR TB treatment organized within the country to another health facility (if patients change address) or to another country?
10. What is the role of General Practitioners / Family doctors in motivating and supporting MDR TB patients to comply with treatment?
11. Health system regulation with regard to TB and MDR TB treatment
12. What regulations/arrangements exist for MDR TB patients to complete treatment in the country even when a patient is undocumented or gets a negative on an asylum request? (probing)
13. Do regulations/arrangements exist on mandatory isolation / admission / treatment and what action is taken if MDR TB patients are unwilling to be treated? (probing)
14. What action is undertaken if there are no treatment options anymore for MDR TB patients (e.g. palliative care, symptomatic treatment)? (probing)
15. Availability and uninterrupted supply of MDR TB drugs
16. Who is responsible for procuring MDR TB drugs in the country
17. Where are MDR TB drugs procured?
18. Is there in-country production of MDR TB drugs?
19. Are MDR TB drugs Quality Assured?
20. Where there MDR TB drug shortages in the last 5 years? (Which drugs, how long). If so how were they solved?
21. Public health information approaches for prevention and control of MDR TB
22. What kind of targeted campaigns for MDR TB have been organized in the past 5 years? What was their focus (medical professionals, patient organisations, etc.) (probing)
23. Assessment of behaviour and attitude towards MDR treatment
24. What personal protection measures do you take to prevent infection with MDR TB bacteria? (probing)
25. What is your (healthcare worker) attitude/feeling towards working with MDR TB patients? (probing)

## Part 2

Open Questions

1. In your opinion, what is/are the main health system factor(s) contributing to successful MDR TB treatment outcomes in your country (maximum three)?
2. In your opinion, what is/are the main health system cause(s) of poor MDR TB treatment outcomes in your country (maximum three)?
3. How do you think treatment outcomes of MDR TB can best be improved in your country?

Most important socio-economic determinants

Please select and put in priority order from the list below the 3 most important individual socio-economic determinants that negatively affect the MDR TB treatment outcome in your country (maximum three)

- Low Socio-economic status
- Low Level of education
- Language barriers
- Cultural barriers
- Long distance to a healthcare facility providing MDR TB treatment support
- Drug addiction
- Alcoholism
- Homelessness
- Other: please specify

Most important service delivery factors

Please select and put in priority order from the list below the 3 most important service delivery factors that positively affect the MDR TB treatment outcome in your country (maximum three)

- Counselling by nurses
- Supervised intake of medicines by health professionals
- Supervised intake of medicines by family members
- Initial hospitalization of patients
- Quality of medicines
- Treatment of side effects
- Provision of Food package / Social system organization
- Defaulter tracing system
- Other: please specify

## Analysis of responses to questions

The responses to questions were analysed and organised following the six building blocks for health systems from ‘Strengthening health systems to improve health outcomes: WHO’s framework for action’: service delivery; health workforce; information; medical products, vaccines and technologies; sustainable financing and social protection; leadership and governance [7] (in brackets question numbers)

**Country’s health system organization and MDR TB situation** (1,8,32)

**Service delivery**

- Diagnosis of MDR and XDR TB (21-23)
- MDR TB treatment (25-26)
- (Duration of) Hospitalisation (2-4,9)
- Teamwork and multidisciplinary teams (24,28-30)
- Cross-border MDR TB case management (12)

**Health workforce**

- Health workforce (5-6,56-57); providing patient-centred services (10,40-46)

**Health information**

- Diversity of MDR TB populations (13-16)
- Information-based decision making (17-20)

**Medical products, vaccines and technologies**

- (Uninterrupted) MDR TB drug supply (27,50-54)

**Sustainable financing and social protection**

- Financing mechanisms for MDR TB services (33-37)
- (Free) Treatment for all MDR TB patients (38-39)

**Leadership and governance**

- Intersectoral collaboration and partnerships (7,11,47-49,55)

Additional information received during interviews beyond the 57 questions was also structured and analysed according to above categories.
